# Progress in Bacteriology

**Published:** 1901-06-29

**Authors:** 


### PROGRESS IN BACTERIOLOGY.

**Organisms resembling the Tubercle Bacillus.**—The property possessed by the tubercle bacillus of resisting the decolorising power of acids, when stained by carbol-fuchsin, was at one time regarded as distinctive. Later it was shown that the leprosy bacillus also behaved in the same way. Welsh[1] gives in detail the various organisms possessing this "acid-fast" property. Some are found in the tissues, secretions and discharges of human subjects; others in domestic animals and in animal food-products; the third variety is saprophytic. Of the first class those found in the respiratory tract are the most important; these are associated with gangrene of the lung (Fraenkel) or are normal inhabitants of the fauces (Zahn). Acid-fast bacilli other than tubercle bacilli are found in some intestinal lesions (Dietrich). The smegma bacillus, although closely resembling the tubercle bacillus, differs from it in being shorter, in being decolorised by alcohol in one minute, and in being nonpathogenic. With regard to the second class, Cowie states that an "acid-fast" organism is normally present on the udders of cows, and many observers have detected a similar microbe in butter. This organism, unlike the tubercle bacillus, grows readily on all media. When inoculated into animals it gives rise to nodular overgrowths. A bacillus of this variety has also been detected in cow-dung (Moeller). Of the saprophytic "acid-fast organisms" the most notable are—firstly, that described by Houston as being present in the effluents from bacterial sewage-beds; and, secondly, a variety which is found on certain grasses, such as Timothy grass. This last variety has been described by Moeller and Lubarsch. From the foregoing it is evident that every care should be exercised to secure the proper recognition of any acid-fast bacillus of a doubtful nature. The crucial test is that of inoculation. Klein[2] states that young cultures of tubercle bacilli sometimes do not exhibit the acid-fast characteristic. He has met with such in horse-serum tubes inoculated from tuberculous guinea-pigs. Klein also offers some further observations on the pseudo-tuberculosis bacillus of Pfeiffer. His specimens were found in the sediment of London drinking water, in sewage, and in six out of 97 specimens of milk. These materials were tested by inoculation of guinea-pigs, rabbits, and mice, the characteristic lesion being local nodular growth with infection of neighbouring lymph glands. The nodules show round-celled infiltration and coagulation necrosis, but no giant cells or typical tubercle bacilli. The pseudo-bacillus on artificial media resembles bacillus coli, and is readily stained by Löffler's blue, by Gram, and by Ziehl-Neilsen's method. Animals infected with the pseudo-tuberculosis bacillus do not react to tuberculin, nor does a true tuberculous infection in any way modify the pseudo-infection.

The tubercle bacillus belongs in all probability to the genus Streptothrix, and in a paper recently read before the Pathological Society of London, Foulerton and Price Jones[3] state that out of ten pathogenic varieties of streptothrix, two (*S. Nocardii* and *capræ*) were both acid-fast and alcohol-fast, whilst two (*S. Eppingeri* and *sabrazi*) were acid-fast only. The streptothrix genus should be classed amongst the higher fungi.

**Yellow Fever.**—Durham,[4] in a preliminary report upon the bacteriology of yellow fever, describes a fine small bacillus which he detected in all the 14 cases examined. In morphology it resembles the influenza bacillus, and is 4 $\mu$ in length. Its presence was detected at post-mortem examinations in all the abdominal viscera, especially in the lower part of the small intestine, and in the mucus pellets in the stools. This organism is probably the same as that described by Sternberg. No other organism was constantly present. The bacillus is very resistant to stains, the most effective being Ziehl's carbol-fuchsin applied for 12 or 18 hours, with subsequent clearance with weak acetic acid. In the tissues it is present in such small numbers that to get good results sedimentation of the juices is necessary. The bacillus of yellow fever does not grow on ordinary media, but by transferring the mesenteric glands to the incubator, and employing an atmosphere of hydrogen, cultures may be obtained. Durham does not credit the possibility of the transference of infection by gnats.

**Hæmorrhagic Infection by a Species of B. mucosus capsulatus.**—Blumer and Laird[5] report a case of septic infection, terminating fatally in 36 hours, due to a variety of B. mucosus capsulatus. The symptoms were: fever, temperature rising rapidly to 103° F., vomiting, purging, cyanosis of hands and feet, collapse. Post-mortem examination showed small hæmorrhages into pericardium, pleuræ, kidneys, spleen, stomach, and intestines. The solitary glands and Peyer's patches were enlarged. The bacilli found in and cultivated from the lung and mesenteric glands were oval in shape, encapsuled, in pairs or short chains or threads, 1 to 4 $\mu$ in length and ·5 in breadth. They showed bipolar staining with Löffler's methylene blue, and were decolorised by Gram. The organism grows well on agar and on gelatin without liquefying. It is non-motile and facultatively anærobic. It forms indol.

**The Bactericidal Properties of Blood.**—The fact that blood and serum have bactericidal properties has been proved by many observers. Buchner attributed this power to the presence of certain ferment-like bodies which he called alexines, whilst Metchnikoff believed that the action was chiefly phagocytic. Wasserman claimed that the bactericidal qualities of blood depended on two constituents, one of which (substance sensibilisatrice) first modified the micro-organisms, whilst the other (alexine) killed it. Whether the bactericidal action of the blood rose or fell during infection has long been a disputed point. Ostrianine[6] from an elaborate series of experiments concludes that there is never any diminution of bactericidal power during infection; generally there is an increase, and as this increase coincides with an increase in the number of leucocytes we may conclude that the bactericidal power is either directly due to the white cells or to substances elaborated by them.

Andrea Ciaccio found that whilst bacteria could be grown in extracts of organs kept at 37° C., yet the juices of the brain, liver, heart, spleen, lungs and muscles of guinea-pigs and other animals had well-marked bactericidal properties, which could be increased on the addition of certain substances such as sodium chloride and dog's serum. He attributes the curative value of raw meat juice as employed in tuberculosis and other diseases to this bactericidal power.

Simon,[7] with a view to ascertaining the origin of the bactericidal substances of the blood, obtained pleural exudate, and by the centrifuge separated the serum from the leucocytes. The leucocytes were then mixed with normal saline, and the various fluids (salt solution, salt solution with added leucocytes, serum, serum with leucocytes) tested with regard to their action upon virulent streptococci. It was found that salt solution alone had but little power of checking the growth of colonies of streptococci, and the same was true of serum without leucocytes. On the other hand, a mixture of salt solution or serum with leucocytes always showed marked bactericidal action, especially against the weaker strains of streptococci. The action is extracellular as well as intracellular.

Wright[8] has recently introduced a method of measuring the bactericidal power of the blood for clinical and experimental uses. Measured volumes of serum and of graduated solutions of serum are introduced into a series of capillary cultivation tubes and then mixed with equal quantities of a gelatin culture containing an appropriate number of bacteria. The tubes are then incubated, and the number of colonies that develop counted under the microscope. Wright found that normal human serum tested with a young typhoid culture showed complete bactericidal action in a twofold dilution, and an almost complete action in ten- and twenty-fold dilutions. Storage of the blood for twenty-four or forty-eight hours, and dilution about 1 in 40 impaired or destroyed the bactericidal action. Addition of anti-typhoid serum also destroyed the action. There is no direct relation between the bactericidal and agglutinating properties of the blood.

[1] The Scot. Med. and Surg. Jour., May, 1901. [2] Rep. of Med. Off. of L. Gov. Brd., 1899-1900. [3] Brit. Med. Jour., May 25, 1901. [4] Bull. Johns Hopk. Hosp., Feb., 1901. [5] Ibid. [6] An. de l'Inst. Past., Ap. 25, 1901. [7] Central. f. Bakt., No. 3 and 4, 1901. [8] Lancet, Dec. 1, 1900.

